# COVID-19 vaccine attitudes among a majority black sample in the Southern US: public health implications from a qualitative study

**DOI:** 10.1186/s12889-022-14905-z

**Published:** 2023-01-12

**Authors:** Wenting Huang, Emily Dove-Medows, Jalissa Shealey, Katia Sanchez, Lauren Benson, DawnSheska D. Seymore, Patrick S. Sullivan, Heather M. Bradley, Aaron J. Siegler

**Affiliations:** 1 Department of Behavioral, Social, and Health Education Sciences, Rollins School of Public Health, Emory University, Atlanta, GA USA; 2 Center for Sexuality and Health Disparities, School of Nursing, University of Michigan, Ann Arbor, MI USA; 3 Department of Population Health Sciences, School of Public Health, Georgia State University, Atlanta, GA USA; 4 Department of Epidemiology, Rollins School of Public Health, Emory University, Atlanta, GA USA; 5 Department of Epidemiology, Georgia State University, Atlanta, GA USA

**Keywords:** COVID-19, Vaccine, Vaccine hesitancy, Health promotion, Health equity

## Abstract

**Background:**

The COVID-19 pandemic continues to have high caseloads in the US, with vaccines a critical component of the response. Disparities in COVID-19 morbidity and mortality have been identified across states and racial/ethnic groups, which are likely in part due to disparities in COVID-19 vaccine uptake. This study aims to better understand and contextualize COVID-19 vaccine hesitancy among persons from under-represented racial/ethnic populations in the Southern US.

**Methods:**

We conducted 29 in-depth interviews with a sample of households in Atlanta, GA that were selected from an address-based sampling frame. We purposively approached households, from February 6 to June 27, 2021, that declined participation in a national COVID-19 serosurvey to gain perspectives of people who are often under-represented in research. Interviews were conducted in-person or over phone calls for participants with that preference. Thematic analysis was used to identify barriers and facilitators of COVID-19 vaccination, and to contextualize drivers of vaccine hesitancy.

**Results:**

Decision-making about vaccination was described as dynamic, and was compared to the feeling of being on a roller coaster. The predominant reported sources of information were mass media and social media. Facilitators of vaccination included altruism, positive communication from trusted community members and workplace colleagues, and local vaccine provision sites. Driving reasons for vaccine hesitancy included limited trust in the government and concerns about COVID-19 vaccine safety, which one participant compared to jumping off a cliff without a tested rope. Among a subset of participants, beliefs regarding perceived intent to harm the Black community were prevalent. Opportunities to optimally address vaccine hesitancy included countering negative social media messages with positive messaging that matches the community’s vivid ways of discussing vaccines, collaborating with community stakeholders on vaccine promotion efforts, and offering workplace-based vaccine promotion efforts.

**Conclusions:**

This study presents data that indicate it may be optimal to more broadly define ‘community’ in COVID-19 vaccine promotion efforts to include social media and workplace venues. To optimize vaccine and vaccine booster uptake and equity, public health must address historic racism and other concerns by using outreach that is grounded in communities.

**Supplementary Information:**

The online version contains supplementary material available at 10.1186/s12889-022-14905-z.

## Introduction

The COVID-19 pandemic first emerged in the United States in 2019, yet cases and hospitalizations continue in 2022 at high rates [1], making vaccination efforts of continuing importance. As of May 18, 2022, there were 82,820,565 reported cases and 998,512 deaths cumulatively nationwide [1], with 1,981,571 cases and 31,794 deaths occurring in the state of Georgia [2]. Along with this substantial toll, racial disparities in COVID-19 infections, hospitalizations, and deaths have been identified nationally, with Black Americans having higher morbidity and mortality compared to non-Hispanic White Americans [3].

Being one of the most successful public health interventions, vaccination prevents 4–5 million deaths every year from a variety of infectious diseases in all age groups [4]. In December, 2020, the United States Food and Drug Administration (FDA) issued an Emergency Use Authorization (EUA) for the first COVID-19 vaccine, and subsequently has provided approval for two COVID-19 vaccines [5]. Currently, COVID-19 vaccines are recommended for persons aged 6 months and older. Boosters are recommended for persons aged 6 months and older. COVID-19 vaccination is the most effective approach to protect against severe illness and mortality.

However, despite success in COVID-19 vaccine development and the great need for vaccination, vaccine uptake rates have been suboptimal. In the US, 78% (258,463,968) have received at least one dose of a COVID-19 vaccine, and 67% (221,190,484) were fully vaccinated as of May 27, 2022 [6]. In Georgia, vaccination rates are lower than the national average, with only 64% (6,667,289) of residents having received at least one dose, and 56% (2,492,281) fully vaccinated as of May 25, 2022 [7]. Given wide, no-cost availability of COVID-19 vaccines, suboptimal vaccine uptake rates are mostly due to vaccine hesitancy [8, 9]. These vaccine uptake rates also indicate racial disparities, with Black people seeming more hesitant to get vaccinated compared to non-Hispanic White and other racial minority groups in most US states, for whom drivers of vaccine hesitancy might be different [10, 11]. In Georgia, more than half (55%) the Black population has not yet received a dose of a COVID-19 vaccine [7].

Disparities in vaccine uptake for Blacks versus Whites have been observed, and this gap has been attributed to historical racism, current discrimination, mistrust of the medical system, and the lack of diverse race representation in clinical trials among Black persons [12–14]. Racial disparities in vaccination indicate different contexts that shape attitudes towards vaccines, suggesting the need for tailored communication and intervention strategies for vaccine and vaccine booster promotion. In addition, Black persons are often under-represented in clinical trials [15]. A study reviewed all COVID-19 vaccine clinical trials found that Blacks represented only 3–10%, while White persons comprised over 80% at all age levels [14]. However, limited studies have examined the context of vaccine attitudes among under-represented racial/ethnic groups in the southern US. This study aims to describe the context surrounding COVID-19 vaccine attitudes among a majority Black sample in order to inform COVID-19 vaccine promotion strategies.

## Methods

This study was part of a national COVID-19 serosurvey in which households were selected with probability sampling from a national address frame that includes nearly all residential addresses in the United States [16]. For the qualitative portion of this study, we sought to gain perspectives of people who are often under-represented in research projects due to not accessing COVID-19 healthcare services or lack of interest in participating in research. We purposively approached households that declined participation in the national serosurvey, and offered them an opportunity to participate in an in-depth interview about participation in COVID-19-related research and attitudes toward COVID-19 vaccines. Recruitment occurred in the metropolitan area of Atlanta, GA, during February 6 – June 27, 2021. This study oversampled predominantly Black and Hispanic census tracts to gain the perspectives of Black and Hispanic individuals. More than half of the interviews (19/29) were conducted prior to when COVID-19 vaccines were available to all residents of Georgia (March 25, 2021). All interviews occurred at a single time point. For all interviews, however, vaccines had been authorized under FDA EUA, and their release to all members of the general public was planned once there was sufficient supply. Teams of three recruiters conducted a door-to-door strategy, approaching selected households during weekend and non-business hours with a verbal offer of study participation. To obtain a diverse sample, we targeted sampling in census tracts with higher minority race/ethnicity concentrations. Recruitment stopped when no new unique themes were identified (data saturation). Eligible participants were 18 or older and willing to complete a verbal consent process. All in-person interviews were conducted outdoors, utilizing recommended social distancing techniques, by trained research assistants with appropriate face mask protection. Participants who preferred to complete a phone interview were provided that option. Each interview was audio recorded and lasted approximately 30–40 min. A compensation of a $50 gift card was provided to each participant.

The interview guide was informed by the Theoretical Domains Framework (TDF) for investigating problems in implementation of health interventions [17]. Focusing on attitudes toward COVID-19 vaccines, the interview used open-ended questions to explore TDF domains including COVID-19 knowledge, perceived benefits and consequences of vaccination at individual and community levels, emotions, trust, social influences, goals/intentions, and action plans [see Additional file 1]. Some interviews occurred during a time in which not all participants were eligible for a COVID-19 vaccine, and interview guides were designed to accommodate this issue.

All interviews were audio recorded and transcribed verbatim. Thematic analysis was used to explore barriers and facilitators of COVID-19 vaccination [18]. Initial codes were deductively generated following the TDF domains in the codebook, and subsequently we developed a series of inductive codes after each team member reviewed at least three transcripts. Each transcript was coded by two independent coders, with discrepancies resolved through discussion or consulting with a third team member. After coding was completed, overarching concepts and themes were identified and discussed by the research team. All transcripts were managed and coded using MAXQDA 2020 (VERBI Software, 2019).

We coded the interview transcripts with TDF domains and inductive codes. However, during the analysis, we identified some themes that were most revealing of participants’ beliefs on COVID-19 vaccination. Therefore, in Table 2, we structured the themes with example quotes, and interpreted the data in light of public health implications for each theme, an approach we have used previously to facilitate the utility of qualitative data for public health practice [19]. The Consolidated Criteria for Reporting Qualitative Research (COREQ) checklist is presented with more details [see Additional file 2].

### Ethical approval

This study was reviewed and approved by the institutional review broad of Emory University (IRB #00000695).

## Results

### Sample characteristics

A total of 262 households were visited by the research team, and 29 participants consented to participate the interview. For the majority of non-participating households, residents were not at home at the time the interview team offered study participation. The 29 interviews participants had an average age of 48.7 years (range 18–77) and nearly half (13/29) were female. The majority were Black/African American (16/29), five were Hispanic/Latinx, two were Asian, and six were White (Table 1). All participants were aware of COVID-19 vaccines and about two-thirds of the participants (20/29) reported willingness to get vaccinated, with 9/20 having received at least one vaccine dose before the interview. Overall, participants willing to receive COVID-19 vaccines perceived it as a protection from a dangerous virus, and believed that vaccination in their community would facilitate a return to “normal”, which was described as life before COVID-19 pandemic.

**Table 1:** Sociodemographic characteristics of participants, February to June 2021 (*N* = 29)

	N (%)
Age (mean, SD)	48.7, 16.7
Sex	
Male	16 (55)
Female	13 (45)
Race/ Ethnicity	
Black	16 (55)
White	6 (21)
Hispanic/Latinx	5 (18)
Asian	2 (6)

### Changing vaccine beliefs

A common theme across all participants was that vaccination beliefs and perceptions were not static. Instead, vaccine beliefs were described as dynamic processes both at individual- and community-levels (Table 2). One participant compared his vaccine opinions to a “roller coaster” that cycled between positive and negative feelings (#5, Black male, age 42). Another participant noted that people in her community were changing their positions over time towards a more favorable attitude on vaccination (#12, Black female, age 55). Another participant described that before making a vaccination decision, “I have to study and get more information” (#8, Black female, age 33).

**Table 2:** Critical themes for COVID-19 vaccine hesitancy with public health implications among Georgia residents, Feburary to June 2021. (*N* = 29)

Themes	Example quotes	Public health implications
Changing vaccine beliefs	Well, to be honest with you, my opinion of it has been somewhat a roller coaster: positive, negative, positive, negative… Kind of middle of the road. (#5, Black male, age 42)	• Vaccine messaging and boosters offers should continue, as people make their decisions over time.
	I think everyone (in my work community) questioned how fast it was made, how safe it would be. But I now see that people are changing their minds and they actually are taking that extra step to go ahead and get vaccinated. (#12, Black female, age 55)	
	I have to read, and I have to study and get more information before I just to and be like “Okay, I want a COVID-19 vaccine.” (#8, Black female, age 33)	
News media and social media as important vaccine information sources	I watch the news a lot. But then I will still research and look things up after I watch the news. (#8, Black female, age 33)	• Facilitate pro-vaccination norms by addressing misinformation and misperceptions on social media. • Increase public health social media presence, emphasizing positive narratives of COVID-19 vaccination success. • Leverage all social media platforms for vaccine messaging, because use is disparate across the platforms (e.g. YouTube, Facebook, WhatsApp).
	I think that sometimes, people are scared because of everything we hear on TV. (#13, Hispanic male, age 54)	
	I watch TV so much, I know everything. I listen to the doctors and the nurses, all the first responders. (#15, Black female, age 76)	
	We’re looking for more data, we’re looking for more information. … None of the stuff that you hear on all the social media. … I get my information basically from the news. (#24, Hispanic male, age 45)	
	You read your Facebook feed or whatever, you’re going to get all ends of the spectrum. So, some people love it. Some people hate it. “It’s the best thing ever and everyone should do it” versus “It’s a tool of the government trying to control our minds” and all points in between. So, I’m nowhere near either end of it. (#2, White male, age 47)	
	YouTube. I don’t think it was any personal experience for them (my friends). It was just this election came and lots of conspiracy theories about everything. (#7, Black male, age 59)	
	A lot of people especially in my community they were very skeptical about the vaccine because of things you hear on the internet and people… I guess their generation (my parents) is influenced a lot by the media…like Facebook, WhatsApp, stuff like that. (#9, Hispanic male, age 39)	
Barriers		
Limited trust in the government	Everyone’s saying different things based on their political view about how effective it they think it is. (#2, White male, age 47)	• Include community in vaccine promotion, making implicitly clear that COVID-19 vaccines are not solely supported by the government. • Promote bipartisan efforts to encourage vaccine access.
	(I trust) The CDC. The government. Well, not the government…Well, the government now…(not) Trump’s administration. (#3, Hispanic female, age 53)	
	Trump. I don’t trust his information… I just don’t trust the things he says because he’ll be rambling. (#8, Black female, age 33)	
	I think people in the ‘60 s were more in tune and more dedicated to keeping the country and each other safe. This time around I don’t feel like it was, that we were all on board with the same stuff. Unfortunately, it’s bad to me but it happened during the political campaigning and stuff. Because I think it was used, both sides instead of more political analyst. When the polio was out, vaccine. I don’t think we were like this one says get it, this one says hold off, this one says it’s not so bad…You just said, “Here’s the vaccine” and people went to get it. (#6, White female, age 77)	
Skepticism about COVID-19 vaccine safety	Frankly, I’m more likely to let someone else be the first person to jump off the high dive to see what it’s like…I’m going to let you make sure that cord bounces back up before I’m willing to strap it around my ankles and take a leap. If it's not effective it's a big waste of time and a bunch of people are in bad shape because of it… Everyone’s saying different things based on their political view about how effective it they think it is. (#2, White male, age 47)	• Disseminate public health messaging from trusted scientific experts and trusted community members. • Provide accurate scientific evidence on vaccine safety and effectiveness. • Emphasize in communication that COVID-19 vaccines and boosters are not new, and they are established and trusted throughout the world.
	Well, there’s just some concern because it was developed so quick. There’s not a lot of information about the differences between the different brands. I’ve read up some things that they’re not really sure about certain side effects. (#5, Black male, age 42)	
	I mean everybody is skeptical about something because it’s new. Because it’s new. It ain’t been tested for five or ten years and you have all these studies like all right. (#4, Black male, age 43)	
	So, this rush job that you got Fauci doing and Trump who was trying to I guess…beat him to the punch, all people that he paid to do it. …they rushing to throw this stuff together and you supposed to be cool with 70 percent and chemicals that I don’t understand the definitions of that I’m throwing in my body. (#10, Black Male, age 40)	
Overgeneralization of anecdotal adverse events and the lack of positive counter-narratives	I was watching something on the news and I think it was a nurse, she got the shot and then she just passed out. That was on TV. It was live. They gave her the vaccine and she was just talking and then she was like “Uh…” and just passed out. I was like “Ah! They’re trying to kill us.” (#8, Black female, age 33)	• Collect and disseminate compelling positive narratives about getting vaccinated and having no/minor side effects. • Clinicians should communicate transparently and clearly about the cause and chance of adverse events.
	I don’t know why different people get reactions…I see that one of the vaccines…on TV, on the news and online, they say that it causes blood clots in certain cases and they stopped manufacturing it…I don’t know which one it was. (#13, Hispanic male, age 54)	
	Well, look what happened this past couple of weeks with the ladies, you know? That was something with the blood clots. That was something that we were like, “oh, snap.” … Not to say that was going to be her (my wife) case, but it could have been. Who knows? (#24, Hispanic male, age 45)	
Conspiracy theories on COVID virus and vaccine intention	I believe that this (COVID virus) is something that was made up in a lab…it might have been meant for us to get this mess… I believe there are forces in the back somewhere that are just taking things the way that they want them to go. I’m just a believer in that. I’ve been studying stuff like that… They’ve got so many conspiracy theories going on now about zombies, and how your heart will stop after about eight months of this vaccine. (#15, Black female, age 76)	• Efforts to counter conspiracy theories must be led by trusted community members. • Acknowledge historic racism as leading to mistrust of current medical system. • Promote trust in modern COVID-19 vaccines and treatment as ways to actively overcome health inequities.
	(I’ve heard that) Bill Gates wants to kill everybody. The vaccine is not for Black people. They want to sterilize all Black people. (#7, Black male, age 59)	
	I was probably talking to my boy…he’s 100 percent against it. He was calling it the poison gas… my wife she’s talking about the news reporter that died in Detroit that took the vaccine. (#10, Black male, age 40)	
	I don’t want to sound crazy, but I really believe in the Zombie Apocalypse and I feel like that’s (vaccine) going to add to the Zombie Apocalypse… Population, population control…They (the government) got to eliminate some of us and they putting something in there (vaccine). (#27, Black male, age 35)	
	When I say control, I mean people getting microchipped. What Bill Gates is creating is a microchip…for the COVID vaccine. If you get the vaccine shot, go home and put a metal thing on where you got your vaccine shot, and I guarantee you the metal thing will stick to you. … That’s a chip to track you and always control you and see where you at and know what’s going on. … It’s something evil. The vaccine is not going to affect people in this generation. It’s for the next generation. It’s going to affect the women that’s going to birth their kids. Something’s going to be in that kid to where they can control something in their brains. (#28, Black male, age 29)	
	I feel like the government is conspiring to control us in any way possible, and I feel like they will do so through controlling the weather, controlling businesses and people… the whole COVID virus is maybe a plan…the vaccine may be something that potentially could affect us in the long run…Whether the full conspiracy is believable or not, every idea derives from fact(s). (#29, Black female, age 23)	
Facilitators		
Positive vaccine promotion from trusted members within the community, such as healthcare workers, political and community leadership, and trusted community members	Because he (my doctor) sat there and gave himself one (flu vaccine) in front of me…The nurse gave him his shot, and then I said, “Okay, come on. Come on with it.” Word of mouth, maybe. I would like to see some of the people that represent us who took the vaccine and nothing has happened to them, they’re in good shape and all of that. (#15, Black female, age 76)	• Emphasizing that trusted members of local and national communities are receiving vaccines may be a promising vaccine promotion strategy.
	When I saw that the numbers were coming down, and I started hearing a lot of positive reports from other people that had gotten it, and my doctor started giving me some positive reports. (#25, Black female, age 60)	
	But my thing is look at who’s getting it (the COVID-19 vaccine). The president is getting it, the vice president, people with influence and money and power are getting it. They’re not actually segregating it and putting it just to low income people. If they did I would think it would be a test. But since majority of people on TV I see doing it so I think it’s a good thing. (#4, Black male, age 43)	
	When I say people I can relate to I’m talking about people in my neighborhood, brothers I know… (describe a person he knows) He stays not far from here. Somebody like him I would trust. (#10, Black male, age 40)	
Mobilizing support for vaccination through work communities	Because she (my sister) works in a bakery and she’s around children…for people to feel safe around her, she had to go ahead and do that (vaccination)…for her, it’s more of a business move, and she’s a conspiracist like me…but her business is more important to her. (#29, Black female, age 23)	• Prioritize vaccine promotion through work communities. • Besides mandatory vaccination programs for workplaces, encourage employers to provide vaccine information, facilitate vaccination experiences sharing and conversations, and offer vaccines on-site.
	I think I heard some of my colleagues at work talking about getting it (COVID-19 vaccine). (#7, Black male, age 59)	
	My community and my neighborhood I wouldn’t know, but my community at my job I think a lot of people were hesitant…But I now see that people are changing their minds and they actually are taking that extra step to go ahead and get vaccinated. (#12, Black female, age 55)	
	I was just iffy about it. … I got it, I’m fine; everyone I work with got it, they’re fine. I haven’t heard any very negative side effects. (#21, White female, age 33)	
Altruistic protection	I was going to take it because I want to be around my mom and dad. (#25, Black female, age 60)	• Public health messaging should emphasize altruistic aspects of vaccination and boosters (e.g. protecting others by reducing the risk of transmitting COVID-19).
	I mean especially the older people. My boss is old, she's old, my mom. It makes me feel good knowing I can't get it and give it to them…I don't mind getting it if it's something that's going to help people feel safe and protect other people. I'm not that selfless. (#21, White female, age 33)	
Providing vaccine access at local and familiar sites at convenient times	(I won’t go to) anything that I have to drive more than ten minutes. My life is around here. I mean, I don't want to drive 30 min to (get COVID-19 vaccine)… my hope is that it will be available in all pharmacies, and then I have one in the corner. (#3, Hispanic female, age 53)	• Increase vaccine access, such as distributing vaccines through neighborhood pharmacies, retailers, churches, recreation centers, and mobile vaccination sites.
	Well, I’ve been going on the site that the governor of Georgia gave for us to go on. But I also take a look at who’s giving it, the different places, like Walgreens, CVS, Sam’s Club, Kroger, so I do look to see other medical offices that are taking appointments. (#12, Black female, age 55)	
	I was with all those people trying to find some place to get it. I was on the phone every day when they had them popping up at the health department. Basically, it was the public health department seemed like the only ones that had it first and then Publix and some of the other Walgreens and stuff started getting it after a month. (#6, White female, 77 years)	

### Sources of vaccine information

Participants felt that news and social media were both important sources of information regarding COVID-19 vaccines (Table 2). Participants described that traditional media, such as television news, were an essential source of learning about vaccines. Only a few participants said they would proactively search government web pages or read scientific journal articles.

Although participants uniformly described distrust of information on social media platforms such as Facebook and YouTube, they frequently referred to stories and opinions that they had encountered through social media as having an important role in shaping their opinions. As one participant noted, “…through your doctor is the most effective way (to get information), but who’s calling their doctor every other day?” (#4, Black male, age 43). Participants recognized that they were frequently exposed to negative information and opinions of COVID-19 vaccines on social media, with one stating, “It’s very easy to spread the negatives. We need to do a better job of spreading the positive news about it, where right now the positive news is being highly outweighed by the negative news.” (#11, White male, age 37).

### Barriers

Four main themes described the barriers to vaccination are: limited trust in the government; skepticism about COVID-19 vaccine safety; overgeneralization of adverse events and the lack of positive counter-narratives; and conspiracy theories on COVID virus and vaccine intention (Table 2).

#### Limited trust in the government

Nearly a third (8/29) of participants voiced concerns about the accuracy of vaccine information provided by the government and/or people with political views differing from their own. One participant noted that the pandemic began during a political campaign season, making some more cautious about sources of COVID-19 vaccine information relative to previous vaccines (#6, While female, age 77). Another participant said that she trusts “The CDC…. not the government…” (#3, Hispanic female, age 53).

#### Skepticism about COVID-19 vaccine safety

When discussing potential consequences of vaccination, participants predominantly focused on vaccine safety rather than on vaccine efficacy. About half of participants (13/29) expressed at least some skepticism about the safety of COVID-19 vaccines, despite some being willing to get vaccinated. Reasons for skepticism included the accelerated development timeline and perceptions that long-term side effects were unknown. Some argued that COVID-19 vaccines have not been tested for “five or ten years” (#4, Black male, age 43) and vaccine ingredients may have unclear effects (#10, Black male, age 40). Similarly, other participants expressed hesitation to be an early vaccine user, with one noting he did not want to be “the first bungee jumper” before making sure “that cord bounces back up” (#2, White male, 47). In the relatively rare instances when participants described vaccine efficacy, most believed that COVID-19 vaccines are largely effective.

#### Overgeneralization of anecdotal adverse events and the lack of positive counter-narratives

A number of participants (5/29) overgeneralized instances of negative side effects, based on singular anecdotes encountered through news or social media. For instance, one participant reported being frightened, thinking “They’re trying to kill us”, when watching a nurse faint after getting a COVID-19 vaccine on live TV, a clip that was widely circulated on social media (#8, Black female, age 33). Some participants were afraid of potential severe side effects such as blood clots, with others tying concerns to pauses in vaccine manufacturing (#13, Hispanic male, age 54 and #24, Hispanic male, age 45).

#### Conspiracy theories on COVID virus and vaccine intention

About one-quarter (7/29) of participants either held or reported hearing “conspiracy theories” regarding COVID-19 and COVID-19 vaccines. Although endorsement of these beliefs was relatively uncommon, these concerns held important sway among those who subscribed to them. Critically, intention to harm Black people was a central part of the narrative for many who held conspiracy theories; this is likely due to historic events and current systemic racism. One participant called COVID-19 vaccines “poison gas” (#10, Black male, age 40), echoing concerns from others that vaccines are intended to “kill everybody” and sterilize all Black people (#7, Black male, age 59). Participants also reported either hearing and believing conspiracy theories that COVID-19 vaccines could lead to a “zombie apocalypse”; could kill people for the purpose of population control; and could monitor next generations with microchips which inserted with vaccines (#15, Black male, age 76; #27, Black male, age 35; #28, Black male, age 29).

### Facilitators

Facilitators of vaccine uptake are identified in Table 2 with public health implications. These include positive vaccine promotion from trusted members within the community; mobilizing support for vaccination through work communities; altruistic protection; and providing vaccine access at local and familiar sites at convenient times.

#### Positive vaccine promotion from trusted members within the community

Participants were motivated to get vaccinated by people they trusted in the community, such as healthcare workers, political leadership, celebrities, and trusted community members. A participant described his previous experience of being motivated to get the flu vaccine by his primary care doctor who “gave himself one (flu shot) in front of me” (#15, Black male, age 76). A participant changed her mind to get vaccinated by seeing positive reports and data about vaccine safety and effectiveness, from both her primary care doctor and people who had been vaccinated (#25, Black female, age 60). Another participant said his concern of vaccine “segregation” was addressed by seeing people in power, such as “the president, the vice president, people with influence and money and power”, getting it (#4, Black male, age 43). Some participants also noted that they would be encouraged to get vaccinated if they heard positive perspectives from their neighbors (#10, Black male, age 40) or positive vaccination experiences among persons from diverse ethnic groups (#15, Black male, age 76).

#### Mobilizing support for vaccination through work communities

Participants felt that work communities could play an important role in mobilizing vaccination. Some participants said that they are not familiar with people or do not feel comfortable discussing vaccination in their neighborhood communities, but were instead more familiar with those they work with. One participant felt social pressure from his racial/ethnic group to not be vaccinated even though he wanted to (#5, Black male, age 42), while another participant mentioned that her sister got vaccinated because of working in a bakery despite having endorsed conspiracy theories (#29, Black female, age 23). Other participants noted that work colleagues were having conversations (#7, Black male, age 59) and encouraging each other to “go ahead and get vaccinated” (#12, Black female, age 55).

#### Altruistic protection

Besides receiving positive support from community, another important motivation of vaccination is the protection of family and friends. A participant said “I was going to take it because I want to be around my mom and dad” (#25, Black female, age 60). Another participant described it as reducing the risk of transmitting the virus to her colleagues and other people, especially the older people: “It makes me feel good knowing that I can’t get it and give it to them…I don't mind getting it if it's something that's going to help people feel safe and protect other people.” (#21, White female, age 33).

#### Providing vaccine access at local and familiar sites at convenient times

About one out of three participants (9/29) who stated an intention to get vaccinated emphasized the importance of local vaccination sites. For instance, one participant was willing to get vaccinated, but only if the site was within a close driving distance: “My life is around here. I don’t want to drive 30 min to (get vaccinated)” (#3, Hispanic female, age 53). Others were looking for vaccine provision at commercially familiar retail locations such as Publix, CVS, and Walgreens, in addition to clinics and other state-run facilities.

## Discussion

### Overview

We identified several major barriers and facilitators in the COVID-19 vaccine decision process at the early stage of vaccine distribution, which provide important context for quantitative data indicating mixed success in vaccine provision efforts. Many of the most common concerns about COVID-19 vaccination can be addressed through effective health communications from and community mobilization by clinicians and public health professionals. For example, public health professionals can (1) recognize the dynamic vaccination decision process and frequently revisit vaccination decisions; (2) explain COVID-19 vaccine development timeline and FDA approval process to validate and address misinformation and misperceptions particularly on vaccine safety for minority populations; (3) facilitate pro-vaccination norms through positive compelling narratives on social media among minority populations, (4) leverage work communities as part of vaccine promotion efforts and emphasize that COVID-19 vaccines will protect everyone; and (5) expand partnerships with pharmacies and retailers to set up vaccination sites to increase geographic accessibility and convenience. We anticipate that our suggestions are also relevant for vaccine booster promotion efforts, because booster shots use ingredients identical in nature to the original vaccines, and even for future vaccine implementation in the next public health emergency.

### Barriers and possible solutions

The major barriers we identified were related to public trust, such as safety concerns fueling by misinformation and “conspiracy theories”. Public trust in the government and public health authorities has previously been identified as a critical component of vaccine confidence [12, 13]. In our study, government mistrust was expressed across all race/ethnicities, even while trust maintained in health authorities such as the CDC, FDA, and their primary care doctors. Participants were skeptical about what has been perceived as accelerated vaccine development and authorization with government and political pressure, and the motives of the government for vaccine promotion. This is concerning because a substantial portion of people with stated willingness to be vaccinated for COVID-19 may not receive vaccines due to these fears [20].

For most participants, vaccine hesitancy was mainly fueling by the concern of vaccine safety. Thus, receiving additional vaccine safety and efficacy information has been identified as a facilitator of vaccine willingness [21]. In our study, vaccine safety concerns, including misinformation and “conspiracy theories”, were brought up more frequently than efficacy as reasons for deciding not to take vaccine, echoing previous study findings in Black and Latino communities [22, 23]. Healthcare providers and public health workers should prioritize addressing the safety concerns by providing scientific data, communicating honestly about anecdotes on the limited vaccine side effects, and disseminating a clear communication about the COVID-19 vaccine development timeline, such as graphic illustrations or brief talking points, while acknowledging historical events that inform current fears around vaccination [24]. In addition, public health workers should recognize the dynamic process of vaccination decisions, revisit people’s decisions and concerns, and emphasize COVID-19 vaccines’ altruistic and individual benefits.

Critically, we noticed that a direct intention to harm Black people was central to the majority of narratives for “conspiracy theories” on the COVID-19 virus and vaccine. These concerns are raised in a background of previous mistreatment of Black persons in the United States, such as the Tuskegee Syphilis Study, which eroded public trust among Black people. As a previous study of racial disparities in influenza vaccination found, Black people had less trust of the government and were more likely to question its motives compared to Whites, which was fueled by historical medical racism and current discrimination [12]. Given the context these “conspiracy theories” are grounded in, it is important to note that although participants called their beliefs “conspiracy theories”, their fears are nuanced in their shared experiences with racism and historical mistreatment. Specific efforts to overcome such concerns must be made to rebuild trust with the medical establishment.

### Facilitators and resource leveraging

In addition to addressing identified concerns, we found that leveraging social media may be a promising avenue to change vaccine hesitancy. Although participants generally did not identify social media as reliable information sources, these social media platforms were frequently discussed when we probed views of vaccines. Previous research found that exposure to favorable comments towards COVID-19 vaccines could lead to more positive vaccine attitudes [25]. However, our study participants pointed out that the impact of negative information could outweighed the positive information on social media. This finding is supported by another qualitative study of Black and Hispanic individuals in New York [26] and a networking analysis with three billion Facebook users [27]. Anti-vaccination views on social media could seriously impede the vaccine uptake. Therefore, public health professionals and community advocates should leverage the power of social media to address misinformation and misperceptions, facilitate positive opinions and post compelling narratives about COVID-19 vaccines. This could be accomplished through collaborations with social influencers and with large advertising campaigns on online platforms.

Our findings indicate that vaccine and vaccine booster advocacy should be supported not only in residential communities, but also in work communities. Community engagement generally focuses on geographically-based and faith-based communities, under the assumption that people are more familiar with their neighbors and fellow parishioners, and therefore, will feel more comfortable with such conversations [28]. Our findings, however, suggest that such an approach may miss a key opportunity to engage people who are less comfortable talking to these groups, as some participants brought up. For such persons, work communities may be a promising alternative source of encouragement. This process has been initiated with the CDC recommending a workplace COVID-19 vaccination program that encourages employers to provide on-site vaccination options at the workplace, and off-site vaccination options in the community [29]. Moreover, some colleges and businesses are requiring students and staffs to be fully vaccinated [30]. Even workplaces that do not mandate vaccination should leverage trusted positions to facilitate positive conversations regarding vaccination and offering vaccination and vaccine boosters on-site.

### Limitations

This study had a number of limitations, including a sample limited to the Atlanta metropolitan area, and some conversations that occurred during a time when not all participants had access to COVID-19 vaccines. The sample in this study only included persons who decided to not participate in a broader serosurvey study. This may introduce bias because groups refusing participating research are more likely to lack healthcare access, endorse medical distrust, and have lower self-efficacy and less social support [14, 31, 32]. Nonetheless, the study has numerous advantages such as inclusion of a diverse population by oversampling under-represented race/ethnicity groups, and a door-to-door sampling strategy that facilitated inclusion of persons who might not traditionally participate in research. Moreover, this study captured important vaccine attitudes in the early stage of novel vaccine promotion.

## Conclusion

Hearing how people give voice to their vaccine support and hesitancy across racial and ethnic groups is critical to optimizing COVID-19 vaccine uptake. Through these in-depth interviews, we identified a number of promising avenues for vaccine promotion. Healthcare providers should address people’s vaccine safety concerns with clinical cases and statistical evidence that are culturally relevant across multiple ethnicities, and recognizing that vaccination decision processes occur over time, and that patients may change their minds over time. Historical events motivating vaccine mistrust should also be acknowledged and addressed. To address predominantly negative messaging, a promising strategy is to disseminate positive narratives about vaccination on social media to counter predominantly negative messaging on such platforms. In addition to health professionals, employers can disseminate vaccine information, facilitate vaccine relevant conversations, and provide on-site vaccination at workplaces when possible. To optimize vaccine uptake and vaccine equity, efforts to support vaccine uptake must continue to be grounded in community-based approaches and actively address concerns that arise from each community.

## Supplementary Information

**Additional file 1.** COVIDVU Interview Guide.

**Additional file 2.** Consolidated criteria for reporting qualitative studies (COREQ): 32-item checklist.

## Data Availability

The datasets used and analyzed during the current study available from the corresponding author on reasonable request.
