# Supplementary material for: COVID-19 vaccine attitudes among a majority black sample in the Southern US: public health implications from a qualitative study
Source: BMC Public Health. 2023 Jan 12;23:88. doi: 10.1186/s12889-022-14905-z (PMC9834032; doi:10.1186/s12889-022-14905-z)
Supplement: Supplementary file 1 — Additional file 1. COVIDVU Interview Guide. [file 12889_2022_14905_MOESM1_ESM.docx]

**Additional file 1: COVIDVu Interview Guide**

(Following consent of participants) Thank you. My name is [name], and I am a researcher at Emory University. Today, we’re going to have a discussion about the COVIDVu study and some related issues about COVID vaccination. This discussion should last about 30 minutes.

Keep in mind that there are no right or wrong answers. The goal of the study is to understand your experience and opinions. If you feel uncomfortable at any time, you can choose not to answer a question or end the interview. Everything that you say here will be kept confidential. The information may be shared with other member of the research staff, but the information you share with me today will only be used for this research project.

If it is all right with you, I would like to audio record our conversation today. This is so that I can remember everything that you say to me, in case I miss something with my notes. The recording will be destroyed once we have reviewed it. Is it okay with you if I record the discussion?

To start, think back to when you first received information about the COVIDVu study (pause briefly):

Q1. What were your thoughts about the study?

Q2. What concerns or hesitation did you have about the study?

Q3. What made you want to participate in the study?

Now, I have some questions about COVID-19 vaccines. This is becoming an important health issue, and we’d like to understand your thoughts.

Q4. (knowledge) What facts do you know about the vaccine?

Q5. (consequences) What do you think would happen if you or someone you know gets the vaccine?

Probe: What about good things that might happen? OR

Probe: What about bad things that might happen?

Q6: (optimism) How will availability of the COVID vaccine influence the amount of COVID disease in your community?

Q7: (emotions): When you think about the COVID vaccine, how does it make you feel?

Probe: What kinds of positive feelings it brings out in you?

Probe: What are some negative feelings it brings out in you?

Q8: (environmental context/social and professional role): Tell me about how where you live and work influences your thoughts on the COVID vaccine.

Q9: (beliefs about capabilities): If you wanted to get the vaccine, do you think you would be able to?

Probe: Why or why not?

Q10: (social influences): How do the people you care about feel about the vaccine?

Q11: (social influences/medical mistrust): Who do you trust to give you accurate information about the vaccine? Who do you not trust to provide accurate information?

Q12: (goals/intention): Tell me about your plans about getting the vaccine.

Probe: What influenced your thoughts on this?

Probe: (Clarify timeline for plans if participant did not make clear)

Q13: (goals/memory, attention, decision) (If participant is planning to get vaccine) Once the COVID vaccine is available, is there anything you can do to help make sure you get it? Is there anything society can do to help make sure you get it?
