# Supplementary material for: COVID-19 vaccine attitudes among a majority black sample in the Southern US: public health implications from a qualitative study
Source: BMC Public Health. 2023 Jan 12;23:88. doi: 10.1186/s12889-022-14905-z (PMC9834032; doi:10.1186/s12889-022-14905-z)
Supplement: Supplementary file 2 — Additional file 2. Consolidated criteria for reporting qualitative studies (COREQ): 32-item checklist. [file 12889_2022_14905_MOESM2_ESM.docx]

Additional file 2. Consolidated criteria for reporting qualitative studies (COREQ): 32-item checklist

| No | Item | Response |
| --- | --- | --- |
| Domain 1: research team and reflexivity | | |
| 1. | Interviewer/facilitator | JS, KS, LB, and DDS collected data. |
| 2. | Credentials | WH, JS, KS, LB, and DDS are MPH. ED, PSS, HMB, and AJS are PhD. |
| 3. | Occupation | WH, JS, KS, LB, and DDS are graduate students. ED is a post-doctoral student. PSS is a professor. HMB and AJS are associate professors. |
| 4. | Gender | WH, ED, JS, KS, LB, DDS HMB are female. PDD and AJS are male. |
| 5. | Experience and training | All interviewers were trained in public health and qualitative data collection via IDIs. All researchers have extensive experiences and trainings in qualitative research. |
| 6. | Relationship established | The research team sent study recruitment letters and COVID-19 home testing kit to study participants’ household prior to the IDIs. |
| 7. | Participant knowledge of the interviewer | Participants were informed about the interviewers’ credentials, affiliations, and reasons for conducting the IDIs. |
| 8. | Interviewer characteristics | All interviewers are interested in the research topic and passionate about collecting qualitative data. |
| Domain 2: study design | | |
| 9. | Methodological orientation and Theory | Thematic analysis was used in this study. Details in the method section. |
| 10. | Sampling | Purposive sampling. |
| 11. | Method of approach | Face-to-face interview. |
| 12. | Sample size | The sample size is 29. |
| 13. | Non-participation | A total of 233 people did not participate in the study. Reasons include refused to participate, no repose, deemed unsafe, and unoccupied household. |
| 14. | Setting of data collection | Outside of participants’ residents |
| 15. | Presence of non-participants | No one present besides the participants and researchers. |
| 16. | Description of sample | Demographic data and interview dates were reported in the result section and method section. |
| 17. | Interview guide | Attached as additional file 1. |
| 18. | Repeat interviews | No repeat interview was carried out. |
| 19. | Audio/visual recording | Audio recording. |
| 20. | Field notes | Field notes were made during the interview. |
| 21. | Duration | 30-40 minutes. |
| 22. | Data saturation | Data saturation was reached when there is no new unique themes were identified. |
| 23. | Transcripts returned | Transcripts were not returned to participants for comment or correction. |
| Domain 3: analysis and findings | | |
| 24. | Number of data coders | There were two coders (WH and ED). |
| 25. | Description of the coding tree | The coding tree was described in the method section. |
| 26. | Derivation of themes | Themes were derived from the data. |
| 27. | Software | MAXQDA 2020 were used to manage the data. |
| 28. | Participant checking | No. Participants did not provide feedback on the findings. |
| 29. | Quotations presented | Quotations were presented to illustrate the themes with participant number, sex, and age. |
| 30. | Data and finding consistent | Yes. |
| 31. | Clarity of major themes | Yes. |
| 32. | Clarity of minor themes | Yes. |
